# Pharmacologic modulation of RORγt translates to efficacy in preclinical and translational models of psoriasis and inflammatory arthritis

**DOI:** 10.1038/srep37977

**Published:** 2016-12-01

**Authors:** Xiaohua Xue, Pejman Soroosh, Aimee De Leon-Tabaldo, Rosa Luna-Roman, Marciano Sablad, Natasha Rozenkrants, Jingxue Yu, Glenda Castro, Homayon Banie, Wai-Ping Fung-Leung, Luis Santamaria-Babi, Thomas Schlueter, Michael Albers, Kristi Leonard, Alison L. Budelsky, Anne M. Fourie

**Affiliations:** 1 Janssen Research & Development, La Jolla, California, United States; 2 Translational Immunology (PCB/UB), Department of Physiology and Immunology, Universitat de Barcelona Barcelona, Spain; 3 Department of Research, Phenex Pharmaceuticals AG, Heidelberg, Germany; 4 Janssen Research & Development, Spring House, Pennsylvania, United States

## Abstract

The IL-23/IL-17 pathway is implicated in autoimmune diseases, particularly psoriasis, where biologics targeting IL-23 and IL-17 have shown significant clinical efficacy. Retinoid-related orphan nuclear receptor gamma t (RORγt) is required for Th17 differentiation and IL-17 production in adaptive and innate immune cells. We identified JNJ-54271074, a potent and highly-selective RORγt inverse agonist, which dose-dependently inhibited RORγt-driven transcription, decreased co-activator binding and promoted interaction with co-repressor protein. This compound selectively blocked Th17 differentiation, significantly reduced IL-17A production from memory T cells, and decreased IL-17A- and IL-22-producing human and murine γδ and NKT cells. In a murine collagen-induced arthritis model, JNJ-54271074 dose-dependently suppressed joint inflammation. Furthermore, JNJ-54271074 suppressed IL-17A production in human PBMC from rheumatoid arthritis patients. RORγt-deficient mice showed decreased IL-23-induced psoriasis-like skin inflammation and cytokine gene expression, consistent with dose-dependent inhibition in wild-type mice through oral dosing of JNJ-54271074. In a translational model of human psoriatic epidermal cells and skin-homing T cells, JNJ-54271074 selectively inhibited streptococcus extract-induced IL-17A and IL-17F. JNJ-54271074 is thus a potent, selective RORγt modulator with therapeutic potential in IL-23/IL-17 mediated autoimmune diseases.

The retinoic acid receptor-related (ROR) sub-family of orphan nuclear receptors^1^ was initially identified on the basis of sequence similarities to the retinoic acid and retinoid X receptor families. Through alternative promoter usage and exon splicing, the ROR genes encode different isoforms of RORα, β and γ, which exhibit differential tissue expression and functions. RORγt is a differentially spliced isoform of RORγ, that differs only in the N-terminus by the presence of 21 additional amino acids in RORγ. The endogenous physiological ligands for RORγt have recently been identified as 7β-27-dihydroxy cholesterol^2^, and two other cholesterol biosynthetic intermediates^3^,^4^.

RORγt is exclusively expressed in cells of the immune system including CD4^+^ CD8^+^ double positive thymocytes^5^, Th17^6^, Tc17^7^, and γδ T cells^8^, as well as a subset of innate lymphoid cells (ILCs)^9^ and regulatory T cells (Tregs)^10^,^11^. RORγt is a key transcription factor driving Th17 cell differentiation, and production of IL-17A, IL-17F and IL-22 in innate and adaptive immune cells, also termed “type 17” cells^12^. Th17 cytokines, IL-17A, IL-17F, and IL-22, stimulate tissue cells to produce a panel of inflammatory chemokines, cytokines and metalloproteases, resulting in the recruitment of granulocytes to sites of inflammation^13^,^14^. The Th17 cell subset has been shown to be the major pathogenic population in several models of autoimmune inflammation, including collagen-induced arthritis (CIA) and experimental autoimmune encephalomyelitis (EAE)^15^,^16^. RORγt deficient mice show impaired Th17 cell differentiation *in vitro*, significantly reduced Th17 cell populations *in vivo*, and decreased susceptibility to EAE^6^ and intestinal inflammation^17^. RORγt-deficient T cells fail to induce colitis in the mouse T cell transfer model^18^.

Human genetic studies have shown association of polymorphisms in the genes for Th17 cell-surface receptors, IL-23R and CCR6, with susceptibility to inflammatory bowel disease (IBD), multiple sclerosis (MS), rheumatoid arthritis (RA) ankylosing spondylitis (AS) and psoriasis^19^,^20^,^21^,^22^,^23^,^24^. Clinical modulation of the IL-23/IL-17 pathway through biologics targeting IL-12/23, IL-23, IL-17A or IL-17RA has provided validation of its critical role in human autoimmune diseases^25^,^26^,^27^,^28^,^29^,^30^,^31^,^32^. RORγt is a nuclear receptor target in the IL-23/IL-17 pathway, and has been shown to be tractable to modulation by oral small molecules^33^, Indeed, other nuclear receptors have been successfully targeted by orally available small molecules that are now marketed drugs^34^.

In this study, we describe a novel, selective and potent RORγt inverse agonist, JNJ-54271074. This molecule specifically blocked RORγt-dependent pathways in cellular assays and significantly reduced inflammation in multiple preclinical and translational models. In particular, we provide the first evidence for oral efficacy of RORγt inhibition in blocking IL-23-dependent psoriasis-like mouse skin inflammation, and inhibition of antigen-triggered IL-17 production in human skin-homing psoriatic T cells. These results provide strong evidence for supporting the potential benefits of therapeutics targeting RORγt in IL-23/IL-17-mediated autoimmune diseases, such as psoriasis.

## Materials and Methods

### One-hybrid reporter assay

The reporter assay was performed by transient co-transfection of HEK293T cells with pCMV-BD (Stratagene #211342) containing the GAL4 DNA-binding domain fused with full-length human RORγt (Genbank accession no. NP_001001523, aa 1–497), pFR-Luc reporter and pRL-CMV reporter (Promega #E2261) plus Carrier DNA (Clontech # 630440) using PEI solution (Sigma Aldrich cat# 40872-7) in a 96-well plate. Cells were incubated for 4–6 hours, and then cultured in MEM supplemented with Glutamax, NEAA, sodium pyruvate and Pen/Strep in the presence of JNJ-54271074 (US9, 290, 476) for 16–20 hours. Medium was removed and cells were lysed with Passive Lysis Buffer (Promega). Firefly luciferase buffer was then added and firefly luciferase luminescence was read on BMG LUMIstar OPTIMA luminescence plate reader. One second later, renilla luciferase buffer was then added and renilla luciferase luminescence was read to measure cell viability. For RORα and RORβ activity, similar assays were performed except cells were transfected with pCMV-BD-RORα (Genbank accession no. NP_599022, aa 305–556) or pCMV-BD-RORβ (Genbank accession no. NP_008845, aa 201–459).

### Fluorescence Resonance Energy Transfer (FRET) co-activator peptide assay

The FRET assay measures the ability of a compound to modulate the interaction between the RORγt ligand binding domain (LBD) and synthetic *N*-terminally biotinylated peptide which is derived from the nuclear receptor co-activator TRAP220. Recombinant 6xHIS-RORγ(LBD) protein, produced in *Ecoli,* was incubated with biotinylated TRAP220(631–655), anti-HIS-Eu-W1024 (Perkin Elmer) as fluorescent donor and SA-APC (Prozyme) as fluorescent acceptor in the presence of DMSO or titrated JNJ-54271074 in a Tris-based buffer system (20 mM Tris-HCl pH6.8; 60 mM KCl, 1 mM DTT; 5 mM MgCl_2_; 35 ng/μL BSA) at room temperature for 1h, then the time resolved FRET signal was measured at 665 nm and 615 nm to calculate activity.

### Two-hybrid NCOR reporter assay

This experimental procedure was similar to the 1-hybrid assay except that HEK293T cells were transiently co-transfected with four plasmids: pCMV-BD fused with NCoR (aa1906-2312); pCMV-AD-RORγt expressing the NFκB-AD-RORγt fusion protein; pFR-Luc reporter and pRL-CMV. 4–6 hours after transfection, different concentrations of JNJ-54271074 were added to the cell culture and incubated overnight. Cells were lysed and luminescence signals were measured as described above.

### Mice

All mice were purchased from Charles River Laboratories (Hollister, CA) except the RORγt heterozygous and homogenous knockout mice and matching wild type mice, which were purchased from Jackson Laboratory (Bar Harbor, Maine). All studies in mice have been carried out in accordance with the animal use guidelines and approved ICAUC protocols by Janssen R&D. LLC.

### Human Samples

Human samples used for this study were provided by various sources as indicated below. Informed consent was obtained from all subjects.

### Antibodies and flow cytometry

The following antibodies (anti-mouse or anti-human) were purchased from BD Biosciences (San Diego, CA) or eBioscience (San Diego, CA): anti-CD3-FITC (mouse 17A2 and human SK7), anti-CD4-allophycocyanin (APC), anti-CD4-PerCp, anti-CD4-FITC, anti-CD4-PE (mouse GK1.5 and human SK3), anti-CD44-PerCp (mouse IM7), anti-γδ TCR-FITC, anti-γδ TCR-PE (mouse GL3 and human B1), anti-CD62L-FITC (mouse MEL-14 and human SK11), anti-CD45RO-APC (human UCHL1), anti-CCR6-PE (human 11A9), anti-CD45-PerCp (mouse 30-F11). Human or mouse anti-IFNγ (mouse XMG1.2 and human 4S.B3), anti-TNFα (mouse MP6-XT22 and human MAB11), anti-IL-17A (mouse eBio17B7 and human eBio64DEC17), anti-IL-22 (mouse 1H8PWSR and human 22URTI), and anti-Foxp3 (mouse FJK-16s and human 36A/E7), all PE or APC conjugated, were purchased from eBioscience (San Diego, CA). Human and mouse APC conjugated CD1d tetramers pre-loaded with alpha-GalCer and negative controls were obtained from ProImmune. For intracellular staining cells were stimulated for 4 hours at 37 °C with PMA and ionomycin (0.5 μg/ml each) in the presence of brefeldin A and monensin or with leukocyte activation cocktail (BD Bioscience, San Diego, CA). Cells were stained with fluorescence-labeled antibodies for surface marker, then fixed/permeabilized (eBioscience) and stained with antibodies for intracellular cytokines. For Foxp3 staining cells were fixed/permeabilized using transcription factor buffer set (eBioscience). The data were acquired on a FACS Calibur (BD Bioscience) and analyzed using FlowJo software (Tree Star, San Carlos, CA).

### *In vitro* mouse Th17 differentiation

Naïve CD4^+^ T cells were isolated from splenocytes of C57BL/6N mice using a naïve CD4^+^ T cell isolation kit II (Miltenyl Biotec, Auburn, CA), following the manufacturer’s instructions. Cells were resuspended in culture medium (RPMI-1640 medium supplemented with 10% fetal bovine serum, 50 IU/ml penicillin, 50 μg/ml streptomycin, 2 mM glutamine, and 55 μM β-mercaptoethanol), and were added to 96-well plates at 2 × 10^5^ per well. Titrated JNJ-54271074 was added to each well at final DMSO concentration of 0.2%. Cells were incubated for 1 hour, after which Th17 cell differentiation medium was added with final concentrations of 1 μg/ml anti-CD3, 5 μg/mL anti-CD28 (BD Pharmingen, San Diego, CA), 10 μg/ml anti-IL-4, 10 μg/ml anti-IFNγ, 10 ng/mL IL-23, 10 ng/mL IL-6, and 10 ng/ml TGFβ (R&D Systems, Minneapolis, MN). Cells were cultured at 37 °C and 5% CO_2_ for 3 days. Supernatants were collected and the accumulated cytokines in culture were measured by ELISA kits (R&D Systems, Minneapolis, MN) following manufacturers’ instructions. Cells were harvested for staining and flow cytometry analysis.

### *In vivo* mouse Th17 differentiation and *in vitro* restimulation

C57BL/6J mice were immunized on day 0 with 20 mg/kg Chicken Ovalbumin (OVA, Sigma) emulsified 1:1 with Complete Freunds Adjuvant, (CFA, Chondrex) and draining lymph nodes (DLNs) were collected on day 14. Single cell suspension was prepared in culture medium and seeded in 96-well plates at 2 × 10^5^ per well. Cells were stimulated with 50 μg/ml OVA in the absence or presence of 1 μM JNJ-54271074 and cultured at 37 °C and 5% CO_2_ for 48 hours, then IL-17A- and IL-22-producing cells were analyzed by intracellular staining and flow cytometry analysis.

### Human memory CCR6^+^ T cells *in vitro* activation

Human blood samples from healthy donors were provided by the Scripps Research Institute, La Jolla following Institutional Review Board approved protocol. Total CD4^+^ T cells were isolated from the peripheral blood mononuclear cells (PBMCs) using a CD4^+^ T cell Isolation Kit II (Miltenyl Biotec, Auburn, CA), following the manufacturer’s instructions. CCR6^+^ cells were isolated from CD4^+^ T cells using phycoerythrin (PE) conjugated anti-CCR6 antibody followed by anti-PE microbeads. Isolated CCR6^+^ T cells were resuspended in culture medium and added to a 96-well plate which was pre-coated with 1 μg/ml anti-CD3 (BD Bioscience) at 1 × 10^5^ cells per well. Titrated JNJ-54271074 was added to each well, then anti-CD28 (eBioscience) was added at final concentration of 5 μg/ml. Cells were cultured at 37 °C and 5% CO_2_ for 6 days. Supernatants were assayed for accumulated cytokines using MSD multi-spot assay (Meso Scale Discovery, Rockville, MD) or R&D ELISA kit, and cells were used for intracellular staining.

### Mouse and human γδ^+^ T cell *in vitro* activation

Mouse γδ^+^ T cells were isolated from splenocytes of C57BL/6N mice using TCR γδ^+^ T Cell Isolation Kit (Miltenyl Biotec, Auburn, CA), following the manufacturer’s instructions. Cells were resuspended in culture medium and seeded in 96-well plates at 5 × 10^4^ per well. JNJ-54271074 was added to each well and incubated for 30 minutes, then IL-1β and IL-23 at final concentrations of 20 ng/ml each and anti-IFNγ at final concentration of 10 μg/ml (R&D Systems, Minneapolis, MN), were added to each well. Cells were incubated at 37 °C and 5% CO_2_ for 65 hours before sample collection. Supernatant samples were collected for measurement of IL-17A, IL-17F and IL-22 using R&D ELISA kits, according to manufacturer’s instructions. Cytokine-producing cells were analyzed through intracellular staining and flow cytometry analysis.

Human γδ^+^ T cells were analyzed only through flow cytometry. Total PBMCs (1 × 10^6^) from healthy donor were cultured in culture medium in 96-well plates. JNJ-54271074 was added to the cells 30 minutes prior to stimulation with Isopentenyl Pyrophosphate (IPP) (InvivoGen, San Diego, CA) plus IL-1β and IL-23 (20 ng/ml each). Cells were incubated at 37 °C and 5% CO_2_ for 7 days, and then processed for intracellular staining.

### Mouse and human NKT cell *in vitro* activation

Single cell suspension was generated from mouse mesenteric lymph-nodes. Cells were re-suspended in culture medium and seeded in 96-well plates at 5 × 10^4^ per well. JNJ-54271074 was added to each well and incubated for 30 minutes, then IL-1β and IL-23 at final concentrations of 20 ng/ml each and anti-IFNγ at final concentration of 10 μg/ml (R&D Systems, Minneapolis, MN), were added to each well. Cells were incubated at 37 °C and 5% CO_2_ for 4 days before analysis. NKT cells were identified among total live cells using anti-CD3 and mouse conjugated CD1d-tetramer pre-loaded with alpha-GalCer, and cytokine-producing NKT cells were analyzed through intracellular staining. For human NKT cells stimulation total PBMCs from healthy donor were cultured in culture medium in 96-well plates at 1 × 10^6^ per well. JNJ-54271074 was added to the cells 30 minutes prior to stimulation with IL-1β and IL-23 (20 ng/ml each). Cells were incubated at 37 °C and 5% CO_2_ for 6 days, and then stained with anti-CD3 and human conjugated CD1d-tetramer pre-loaded with alpha-GalCer. Cytokine-producing NKT cells were analyzed through intracellular staining.

### Mouse collagen-induced arthritis model

Mice (female DBA/1Lacj mice, Jackson Laboratories; 8–10 weeks) were housed with 12 hours light-dark cycle with food and water provided *ad libitum*. Following acclimation, mice were immunized with an emulsion containing equal amounts of Chick Type II Collagen (Chondrex, Redmond, WA) and Complete-Freund’s adjuvant (CFA, Chondrex), 100 μg each per mouse, on day 0 at the base of the tail and administered a second immunization boost on day 21. On day 21, animals were examined for clinical arthritis scores and randomized into treatment groups. JNJ-54271074 (0.3, 3, 10, 30, or 60 mg/kg) or vehicle (20% HPCD) were given orally, twice-daily (8 hour apart) from day 21 to 35. The clinical arthritis scores were evaluated from days 21 to 34. Animals were euthanized under CO_2_ on day 35. Both hind-paws from each animal were collected and fixed in 10% neutral buffered formalin for histopathology. Both front paws were digested after removing the skin in 0.125% (v/v) Dispase II (Roche, Indianapolis, IN), 0.2% collagenase II (Roche, Indianapolis, IN), and 0.2% collagenase IV (Sigma-Aldrich, St. Louis, MO) in HBSS for 75 min at 37 °C and passed through a cell strainer. Cells were then stimulated and analyzed by flow cytometry for cytokine-producing cells.

### IL-17A production from PBMC of healthy and rheumatoid arthritis patients

Frozen PBMCs of healthy and rheumatoid arthritis patients were obtained from SeraCare Life Sciences (Gaithersburg, MD). Cells were cultured at 3 × 10^6^/ml in culture medium in the presence or absence of JNJ-54271074 under neutral activation (anti-CD3/CD28 beads, Miltenyl Biotec,) or Th17 condition (anti-CD3/anti-CD28 beads, 10 ng/ml of IL-1β, and 10 ng of IL-23 from R&D). 3 days later, supernatants were collected and analyzed by MSD multi-spot assay or ELISA for IL-17A, IL-22 and IL-13 production.

### IL-23-induced skin inflammation

RORγt^−/−^, and RORγt^+/−^ and C57BL/6J wild type mice (The Jackson Laboratory) were injected intradermally with 10 μl of IL-23 (500 ng, R&D Systems, Minneapolis, MN) or 0.9% NaCl on each ear for 7 days. For the JNJ-54271074 study, C57BL/6N mice (Charles River) were dosed orally BID with compound or vehicle control (20% 2-hydroxypropyl-β-cyclodextrin) for 7 days and on day 8 animals were euthanized with CO_2_ and ear tissues were collected for histology and gene expression. Single cell suspension was prepared by digesting the ears using 1 mg/ml collagenase D (Roche, Indianapolis, IN) for 2 min, then stimulated and analyzed by flow cytometry for cytokine-producing cells.

### Histology

Tissues were fixed in 10% formalin, sectioned, and stained with H&E for histology at Seventh Wave Laboratories, LLC, (Chesterfield, MO).

### RNA extraction, quantitative RT-PCR

Total RNA was extracted from the ears using RNeasy plus mini kit (Qiagen). The concentration was measured using NanoDrop ND-1000 Spectrophotometer (Thermo Fisher Scientific, Wilmington, DE). RNA was converted to cDNA using high capacity cDNA Reverse Transcription kit (Life technologies, Foster City, CA) and real time PCR reaction was performed using Quant Studio 12k Flex (Life technologies). All Taqman probes were purchased from Life technologies, mIL-17A Mm00439619_m1; mIL-17F Mm00521423_m1; mIL-22 Mm00444241_m1, IFN-γ Mm00801778_m1; IL6 Mm00446191_m1; TNFα Mm00443260_g1; B2M Mm00437762_m1.

### Co-culture of human psoriatic CLA^+/−^ memory T cells with epidermal cells

The study protocol was reviewed and subsequently approved by the Medical Ethics Committee of the Hospitals where the biopsies and blood were procured. All psoriasis donors in this study were required to be without disease-specific systemic therapy for at least 4 weeks or topical treatment for at least 2 weeks before the clinical samples were obtained. Circulating CLA^+^/CLA^−^ CD45RO^+^ CD3^+^ cells and lesional epidermal cells were obtained from the same guttate psoriasis patient following the procedure described previously^35^. In brief, memory T cell populations were isolated and enriched from peripheral blood lymphocytes by immunomagnetic separations. Epidermal cell suspensions were obtained from lesional biopsies by a dispase/trypsin treatment. The culture system was performed by seeding 50,000 circulating CLA^+^ or CLA^−^ memory T cells with 30,000 autologous epidermal cells in a 96-flat bottom microwell plate (Nunc, Roskilde, Denmark) with culture medium (RPMI with 10% fetal calf serum). Cultures were activated by the addition of Streptococcal extract (1 μg/ml final) isolated from bacteria from the throat of guttate psoriasis patients and incubated for 5 days in the presence of 1 μM JNJ-54271074. Cytokines in supernatants were quantified by multiplex fluorescent bead-based immunoassay, Diaclone DIAplex kit (Gen-Probe, Besançon, France), and data were collected with a F500 Flow Cytometer (Beckman Coulter, Fullerton, CA).

## Results

### Identification of selective RORγt inverse agonists

**Figure 1:**
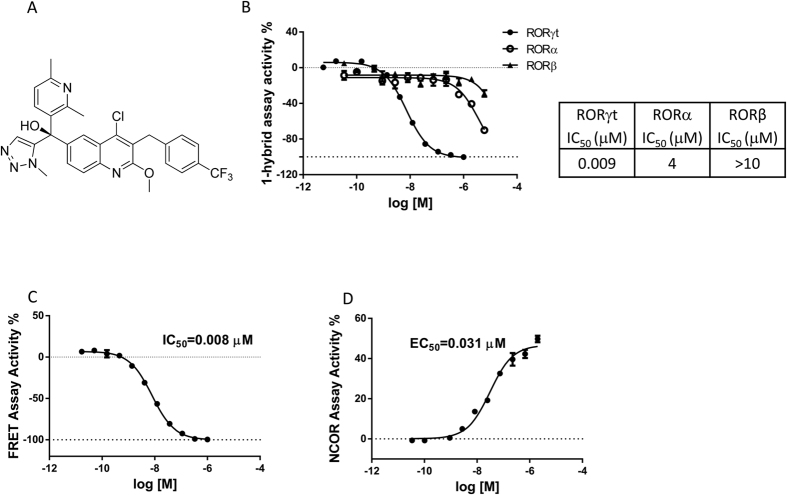
(**A**) Structure of JNJ-54271074. (**B**) Activity of JNJ-54271074 in 1-hybrid reporter assays. HEK-293T cells were transfected with RORγt, RORα and RORβ, which were fused with GAL4 DNA binding domain. After incubation with compound overnight, luciferase signals were measured. JNJ-54271074 was tested at a starting concentration of 1 μM for RORγt, and 6 μM for RORα and RORβ in 3-fold serial dilutions in duplicate. (**C**) FRET co-activator peptide assay. RORγt LBD was incubated with biotinylated TRAP220(631-655) as well as fluorescent donor and acceptor in the presence of titrated JNJ-54271074 and signals were measured at 665 nm and 615 nm (**D**) 2-hybrid co-repressor reporter assay. HEK-293T cells were transfected with NCoR and RORγt that was fused with NFκB activation domain in the presence of titrated JNJ-54271074. NCoR binding with RORγt triggers NFκB –driven luciferase signal. Representative dose response curves (of more than 30 experiments for the 1-hybrid assay, 4 experiments for the FRET assay and one experiment for the 2-hybrid assay) were plotted by percent activity of DMSO control vs. different concentrations, and presented as mean ± SEM of triplicate assays.

An initial high-throughput screen (HTS) of approximately 300,000 proprietary compounds was performed using the ThermoFluor^®^ assay that measures binding to the human RORγt ligand binding domain (LBD) as a function of thermal stabilization^2^. Several chemotypes were identified that bound to the RORγt LBD, and demonstrated dose-dependent functional inhibition of RORγt in cell-based reporter assays. JNJ-54271074 (US9, 290, 476) was developed through optimization of quinoline tertiary alcohol HTS hits, and the chemical structure is shown in Fig. 1A. In the 1-hybrid reporter assay, JNJ-54271074 showed potent, dose-dependent inhibition of RORγt-driven transcription, with an IC_50_ of 0.009 μM. In comparison, IC_50_ values for RORα and RORβ were 4 μM and >10 μM, respectively, in similar reporter assays (Fig. 1B), indicating high selectivity for RORγt.

### Impact on co-activator and co-repressor binding with RORγt

Nuclear receptors interact with co-activators or co-repressors to regulate gene transcription^36^. The effects of JNJ-54271074 on the interaction of RORγt with co-regulators were therefore examined. In a FRET-based assay that measures the binding of RORγt to a co-activator peptide, JNJ-54271074 demonstrated dose-dependent inhibition of the FRET interaction signal with an IC_50_ of 8 nM, indicating that JNJ-54271074 could potently inhibit co-activator binding to RORγt (Fig. 1C).

A two-hybrid NCoR assay was used to measure the impact of JNJ-54271074 on RORγt interaction with a co-repressor domain in a cellular system. In this assay, compounds that increase binding of RORγt (NFκB-AD-RORγt protein) to NCoR (co-repressor domain) result in increased NFκB transcriptional activation of the GAL4 promoter and a resulting increase in luciferase expression. JNJ-54271074 showed a dose-dependent increase of luciferase signal with an EC_50_ of 30 nM, indicating that it could potently promote co-repressor binding to RORγt (Fig. 1D).

### Inhibition of mouse Th17 cell differentiation and IL-17A production *in vitro*

**Figure 2:**
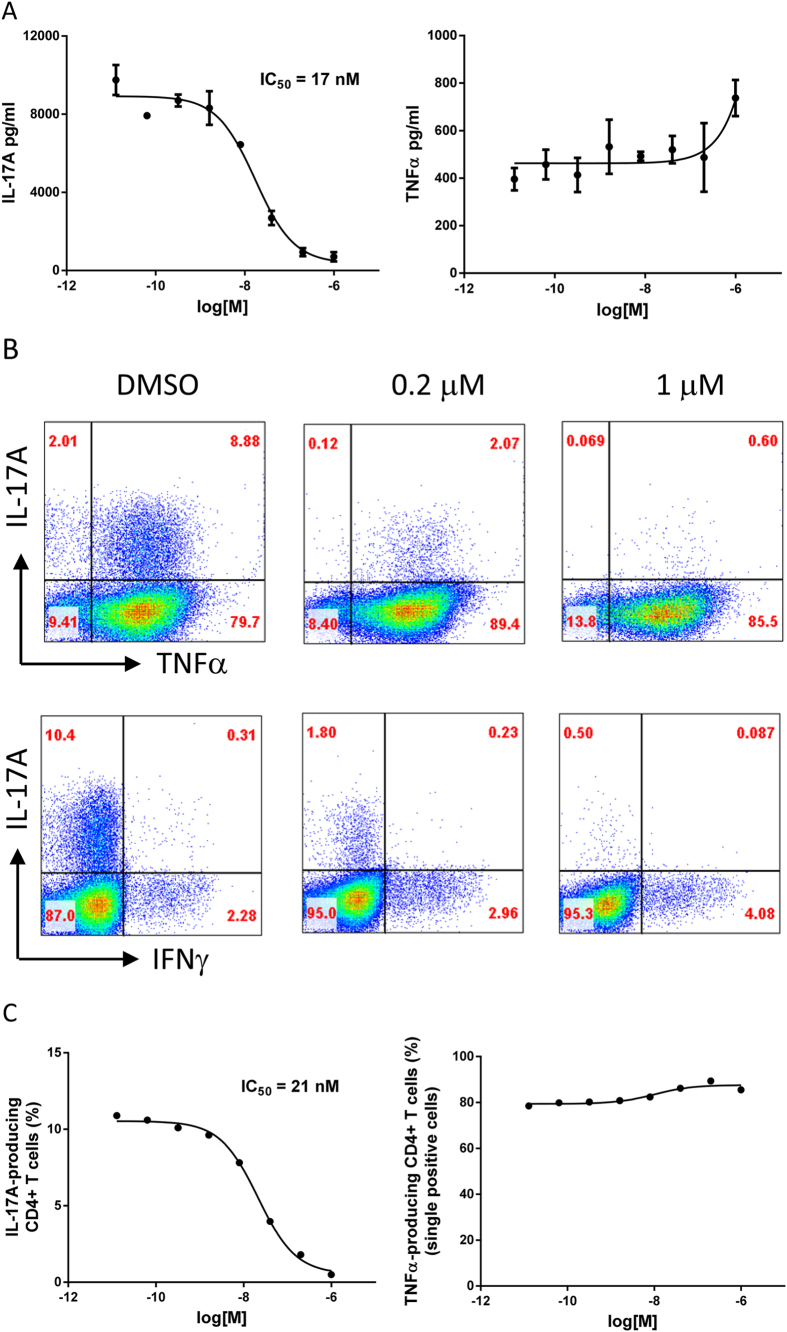
JNJ-54271074 blocked mouse Th17 differentiation and IL-17A production *in vitro*. Pooled purified naive CD4^+^ T cells from spleens of four C57BL/6 mice were cultured under Th17-polarizing condition for 3 days in the absence (DMSO control) or presence of titrated JNJ-54271074 (starting at 1 μM and 5-fold serial dilution in duplicates). (**A**) Accumulated IL-17A and TNFα in cell culture supernatants. Representative data from one of 3 experiments are presented as mean ± SEM of duplicate samples. (**B**) Flow cytometry analysis of IL-17A-, TNFα- and IFNγ-producing CD4^+^ T cells for DMSO and JNJ-54271074 at 0.2 μM and 1 μM. Pooled duplicate samples were used for analysis. (**C**) Dose response curve of JNJ-54271074 on IL-17A- and TNFα-producing CD4^+^ T cells.

The effects of JNJ-54271074 on Th17 differentiation were investigated using naïve CD4^+^ T cells isolated from mouse splenocytes, cultured under Th17-polarizing conditions in the presence of JNJ-54271074. On day 4, supernatants were analyzed for IL-17A and TNFα and cytokine-producing CD4^+^ T cells were analyzed by flow cytometry. JNJ-54271074 dose-dependently suppressed production of IL-17A with an IC_50_ of 17 nM (Fig. 2A). However, JNJ-54271074 treatment did not inhibit TNFα production, and slightly increased TNFα levels at higher concentrations. JNJ-54271074 almost completely abolished IL-17A-producing cells at 1 μM, with a clear dose-dependence between 1 μM and 0.2 μM (Fig. 2B). Quantitative analysis of total IL-17A-producing cells at different concentrations of JNJ-54271074 generated an IC_50_ of 21 nM (Fig. 2C). Approximately 80% of IL-17A-producing cells were also TNFα positive, and IL-17A production from this double-positive population was decreased by JNJ-54271074 (Fig. 2B). There were slightly more TNFα single-positive cells with compound treatment compared to control (Fig. 2B and C) and this may explain the observation of a slight increase in TNFα concentrations in cell supernatants observed at high concentrations of JNJ-54271074. Although there were very few IL-17A/IFN-γ double positive cells detected in these cell cultures, JNJ-54271074 decreased this population as well, which may have contributed to the slight increase in IFNγ single-positive cells.

To determine the specificity of RORγt modulation on T helper cell lineages, we set up both Th17 and Th1 polarization conditions in parallel using isolated mouse CD4^+^ T cells. JNJ-54271074 demonstrated dose-dependent inhibition of IL-17A production as expected under Th17 conditions, while in contrast, minimal effects on IFNγ production were observed under Th1 conditions (Supplementary Figure 1A and 1B). IL-23-induced production of IL-17A and IL-22 in mouse splenocytes in the absence of anti-CD3/anti-CD28 stimulation was also examined. Although IL-17A and IL-22 concentrations were lower than what we typically observed under stimulation conditions with anti-CD3/anti-CD28, JNJ-54271074 demonstrated a clear, dose-dependent reduction in IL-23-stimulated IL-17A and IL-22 production with IC_50’s_ of 19 nM and 37 nM, respectively (Supplementary Figure 2A and B).

### Inhibition of Th17 cytokine production by effector/memory CD4^+^ T cells

**Figure 3:**
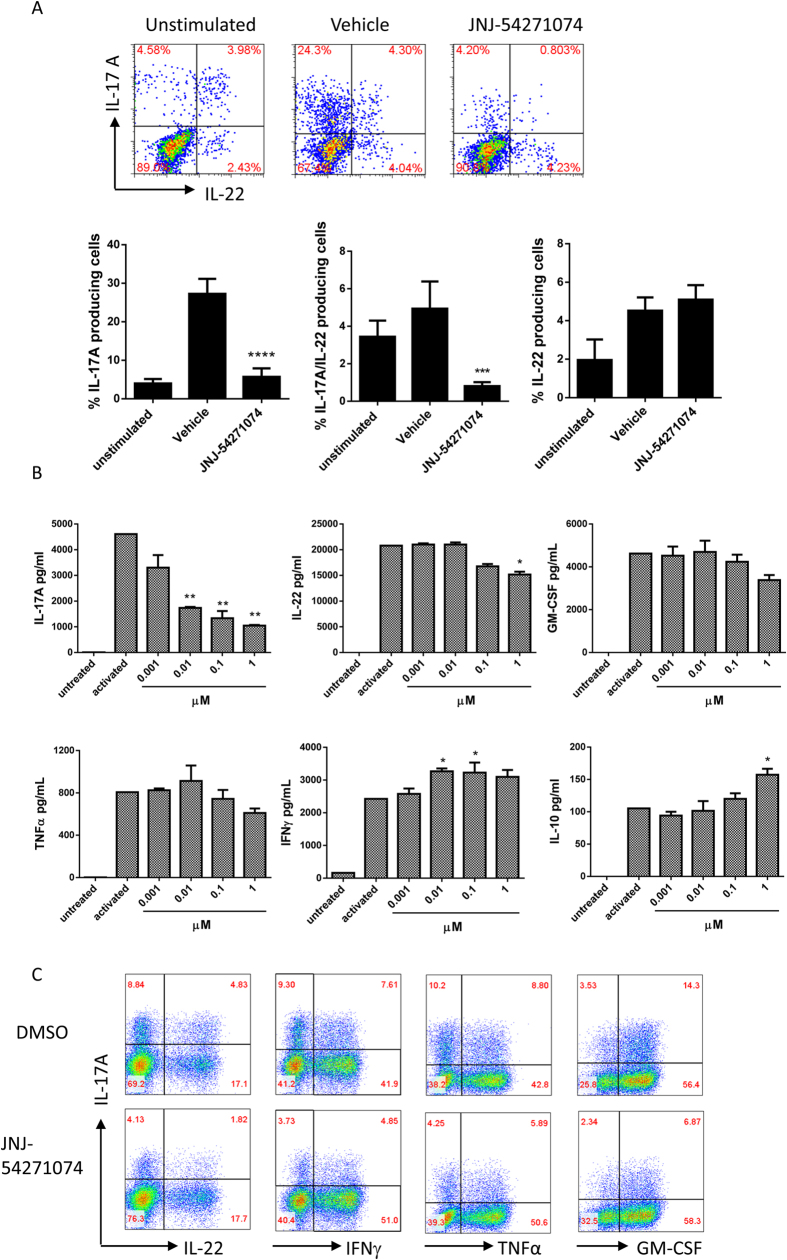
JNJ-54271074 suppressed mouse and human memory T cells’ production of Th17 cytokines. (**A**) CD4^+^ T cells isolated from DLNs of the mice that were immunized with OVA/CFA were re-stimulated with OVA *in vitro* in the presence and absence of 1 μM JNJ-54271074. CD4^+^ CD44^+^ cells were gated and analyzed for IL-17A- and IL-22- producing cells by flow cytometry. Results are presented as mean ± SD of four individual mice and are representative of two independent experiments. (**B**) Human CCR6^+^ cells were purified from CD4^+^ T cells isolated from blood of a healthy donor, and stimulated with anti-CD3/CD28 beads in the absence or presence of titrated JNJ-54271074 for 6 days. Accumulated cytokines, IL-17A, IL-10, IFNγ, TNFα and GM-CSF were measured by MSD, and IL-22 by ELISA. Data are representative of two independent experiments and presented as mean ± SEM of duplicate assays. All the statistical analyses were performed using one way ANOVA Dunnett’s test, *P < 0.05; **P < 0.01; ***P < 0.005; ****P < 0.0001 (**C**) Cells treated with DMSO or 1 μM JNJ-54271074 from the same experiment were used for flow cytometry analysis for IL-17A-, IL-22-, IFNγ-, TNFα- and GM-CSF- producing human CD4^+^ T cells.

Since the majority of CD4^+^ T cells producing IL-17A under inflammatory conditions are effector memory T cells, we evaluated whether RORγt inhibition could suppress IL-17A production by *in vivo* differentiated Th17 cells. We examined the effect of RORγt inhibition on mouse *in vivo* differentiated, OVA-specific effector Th17 cells. CD4^+^ T cells from draining lymph nodes (DLNs) of *in vivo* OVA/CFA immunized mice were restimulated *in vitro* with OVA, and analyzed by flow cytometry. Upon restimulation, increased IL-17A- or IL-22-producing cells were observed compared to unstimulated cells. The addition of JNJ-54271074 significantly suppressed the increased IL-17A^+^ and IL-17A^+^ IL-22^+^ CD4^+^ T cells, with no effect on IL-22 single positive cells (Fig. 3A).

We also examined the effect of JNJ-54271074 on human effector/memory T cells, since this population is a significant source of IL-17A and other inflammatory cytokines^37^. Human CD4^+^ CD45RO^+^ CCR6^+^ T cells were isolated from blood-derived memory CD4^+^ T cells, and cultured for 5 days in the presence of JNJ-54271074 under non-polarizing activation conditions, after which accumulated cytokines in the culture supernatants were measured. JNJ-54271074 significantly and dose-dependently inhibited IL-17A production compared with the DMSO vehicle control, reaching 77% inhibition at 1 μM (Fig. 3B). The same concentration of JNJ-54271074 showed a trend of decreasing IL-22, TNFα and GM-CSF production, while slightly increasing IFNγ production. In addition, 1 μM JNJ-54271074 significantly increased IL-10 production by 49% over the control. Cytokine-producing cells were analyzed by flow cytometry and JNJ-54271074 significantly decreased the number of IL-17A-producing cells, including IL-17A single-positive cells, IL-17A/IL-22, IL-17A/IFNγ, IL-17A/TNFα and IL-17A/GM-CSF double-positive cells, respectively (Fig. 3C). There was minimal effect on IL-22 and GM-CSF single-positive cells, and some increase in IFNγ and TNFα single-positive cells. We also tested the effect of JNJ-54271074 on these memory cells that were cultured without activation, and we observed similar inhibition of IL-17A single-positive cells and IL-17A/IL-22 double-positive cells (Supplementary Figure 3A).

The effects of JNJ-54271074 were also examined on CD4^+^ CD45RO^+^ CCR6^+^ T cells under Th1 polarization conditions in the presence of JNJ-54271074. The compound slightly increased IFNγ^+^ cells, but decreased IL-17A^+^/IFNγ^+^ and IL-17A^+^ cells, with minimal effect on accumulated IFNγ production (Supplementary Figure 3B and C).

RORγt is expressed in a subset of regulatory T (Treg) cells^38^, and therefore the effect of JNJ-54271074 on Treg differentiation/expansion was also examined. CD4^+^ T cells isolated from human blood were cultured under Treg polarizing conditions for 6 days in the presence of JNJ-54271074. Both percentage of FOXP3^+^ T cells and FOXP3 mRNA expression increased under Treg polarizing conditions compared to neutral activation conditions. JNJ-54271074 had no impact on the percentage of FOXP3^+^ T cell, but slightly increased mRNA expression of FOXP3 (Supplementary Figure 4A and B). We also evaluated the effect of JNJ-54271074 on the function of inducible regulatory T (iTreg) cells. Treg cells were generated from human naïve CD4^+^ T cells after 7-day culture in the presence of JNJ-54271074, and then co-cultured with responder CD4^+^ T cells. The proliferation measured by FACS for CFSE staining showed Treg suppressive activity on T cell proliferation was not affected (Supplementary Figure 4C).

### Inhibition of IL-17A production by mouse and human innate like T cells: γδ and NKT cells

**Figure 4:**
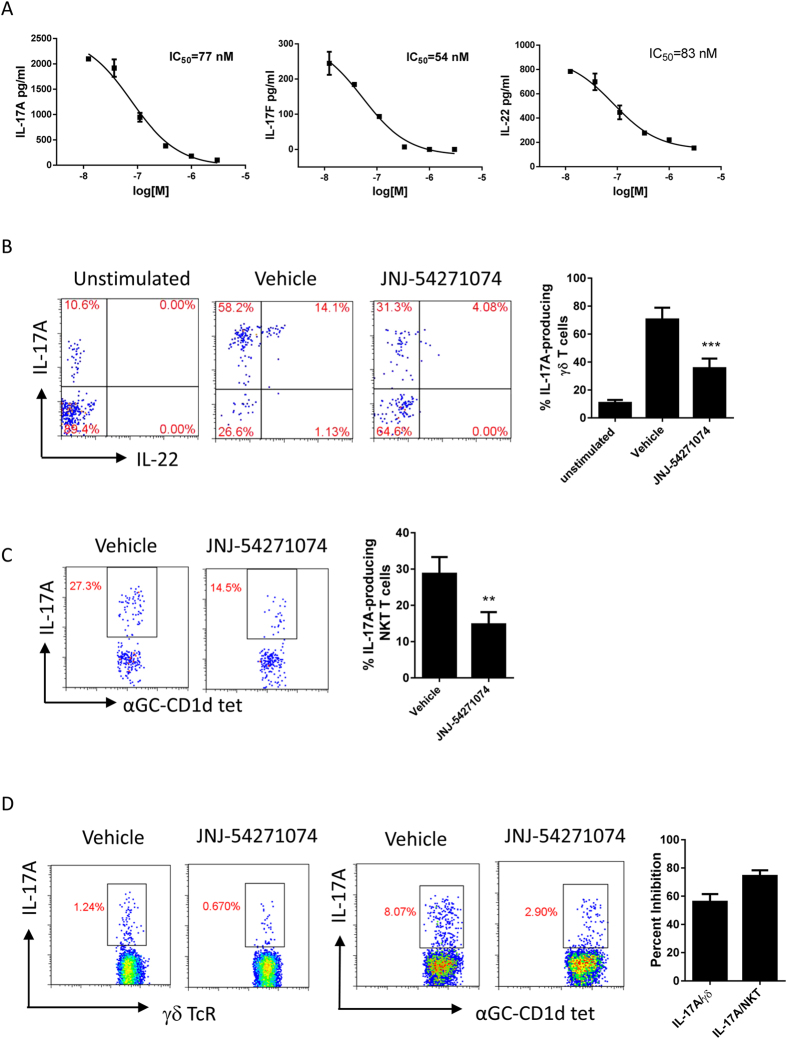
The effect of JNJ-54271074 on mouse splenic γδ T and NKT cells or human peripheral γδ T and NKT cells. (**A**) Dose-dependent inhibition of production of IL-17A, IL-17F and IL-22 in mouse γδ T cells. γδ T cells were isolated separately from four mouse spleens and cultured under stimulation of IL-1β/IL-23 plus anti-IFNγ for 65 hours, in the absence or presence of titrated JNJ-54271074, and accumulated cytokines were measured by ELISA. Data are presented as mean ± SEM of duplicate assays. (**B**) Flow cytometry analysis for mouse IL-17A- and IL-22-producing γδ T cells, is shown as representative FACS plots from one individual mouse and mean ± SD of four mice is shown in the graph. Intracellular staining was performed on unstimulated, DMSO or 0.1 μM JNJ-54271074-treated cells. (**C**) Flow cytometry analysis for IL-17A- producing mouse iNKT cells. Splenocytes from 4 individual mice were stimulated with IL-1β/IL-23 plus anti-IFNγ in the absence or presence of 1 μM JNJ-54271074 for 4 days. IL-17A-producing NKT cells were analyzed through intracellular stain on NKT cells that were identified using anti-CD3 and mouse conjugated CD1d-tetramer pre-loaded with alpha-GalCer. Results are shown as representative FACS plots from one individual mouse and graphed as mean ± SD of four mice, and are representative of two independent experiments (**D**) Flow cytometry analysis for IL-17A- producing γδ^+^ T cells and IL-17A- producing iNKT cells in human PBMCs. PBMCs isolated from healthy donors were stimulated with either IL-1β/IL-23/IPP for 7 days (γδ^+^ T cells) or IL-1β/IL-23 for 6 days (iNKT cells), in the absence or presence of 1 μM JNJ-54271074. Results are shown in a histogram as the percent inhibition relative to vehicle and presented as mean ± SD of three to four different donors. The statistical analysis was performed using one way ANOVA Dunnett’s test for Figure B and unpaired t test with Welch’s correction for Figure (**C** and **D**), *P < 0.05; **P < 0.01; ***P < 0.005.

While IL-17A is widely described as a CD4^+^ Th17 cell secreted cytokine, a large amount of IL-17A can be produced by innate immune cells during an inflammatory response. Mouse γδ T cells and invariant NKT cells are considered major innate sources of IL-17A and IL-22 during autoimmune inflammation^39^,^40^,^41^. Thus, we investigated the effect of JNJ-54271074 on these cell populations. γδ T cells were isolated from mouse spleens and stimulated with IL-1β and IL-23 in the presence of JNJ-54271074. Dose-dependent inhibition was observed for IL-17A, IL-17F and IL-22 production with IC_50_ values of 77 nM, 54 nM and 83 nM, respectively (Fig. 4A). The combination of IL-1β and IL-23 induced significant numbers of IL-17A- and IL-17A/IL-22-producing γδ T cells in comparison to untreated cells, and JNJ-54271074 at 0.1 μM decreased the percentage of both IL-17A- and IL-17A/IL-22-producing cells by about 50% (Fig. 4B).

In contrast to mouse γδ T cells, human peripheral blood γδ T cells do not produce IL-17A *in vitro* in response to IL-1β and IL-23 alone. However, activation of peripheral blood Vγ9Vδ2 T cells with IPP in the presence of Th17 differentiation cytokines leads to induction of IL-17A^42^. We therefore analyzed IL-17A^+^ cells in populations of human CD3^+^/γδ^+^ cells stimulated with IL-1β, IL-23 and IPP, and observed that JNJ-54271074 inhibited the IL-17A^+^ γδ^+^ T cell population by average of 56% (Fig. 4D).

Mouse and human iNKT cells produce IL-17A when stimulated with IL-1β and IL-23^43^,^44^. Similarly to γδ T cells, JNJ-54271074 suppressed IL-17A production by mouse iNKT cells from mesenteric lymph-nodes (Fig. 4C), and by iNKT cells from human PBMCs (Fig. 4D).

### Attenuation of inflammation in a mouse collagen-induced arthritis model

**Figure 5:**
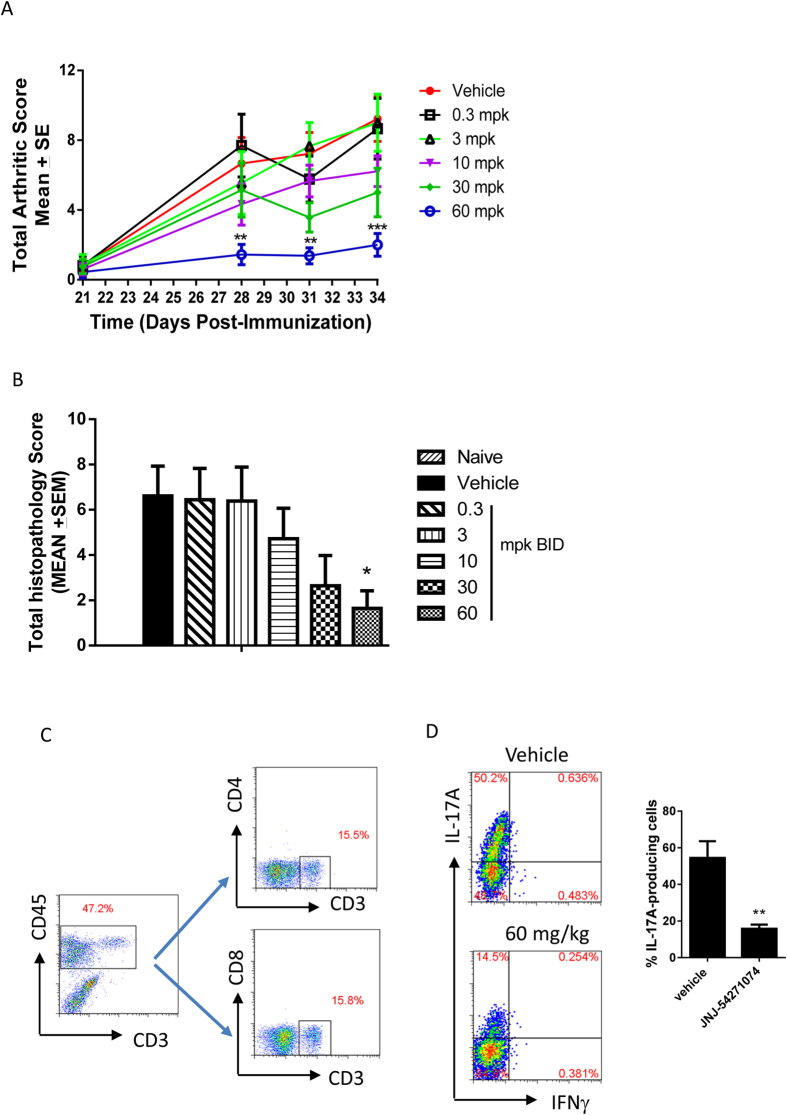
JNJ-54271074 attenuated the development of collagen-induced arthritis (CIA) in mice. (**A**) Time course of CIA clinical score following treatment with vehicle or JNJ-54271074 (0.3, 3, 10, 30 and 60 mpk BID). Clinical scores were monitored on day 21, 28, 31 and 34 after the immunization (n = 8-10 mice per group). Values are the mean ± SEM. **p < 0.01, ***p < 0.005 versus vehicle by one way ANOVA (Dunnett’s multiple comparisons test). (**B**) Total histopathology score of four pathology indices (inflammation, cartilage damage, bone destruction and remodeling) in hind paws, harvested on day 35 (n = 14-18 paws per group). Values are the mean ± SEM. *p < 0.05 versus vehicle by one way ANOVA (Bonferroni’s Multiple Comparison Test). (**C**) Flow cytometry analysis on CD3^+^ T cell population in paws on day 35. Front paws were digested, and cells were harvested and pooled for staining. (**D**) Flow cytometry analysis of CD3^+^ T cells in front paws for IL-17A- and IFNγ- producing cells. Both FACS plots are representatives from one mouse and the % IL-17A-producing cells are also shown in histogram as mean ± SD of four mice. The results are representative of three independent experiments. The statistical analyses were performed using one way ANOVA Dunnett’s test, *P < 0.05; **P < 0.01; ***P < 0.005.

To investigate the role of RORγt in innate and adaptive immune responses *in vivo*, we examined the effects of orally administered JNJ-54271074 in a mouse collagen-induced arthritis model. Treatment with JNJ-54271074 starting on day 21 decreased arthritic scores in a dose-dependent manner (Fig. 5A). On day 34, mice treated with 10, 30 and 60 mg/kg BID doses showed a reduction in the total arthritic scores of 32%, 46% and 78%, respectively, relative to vehicle. At 60 mg/kg BID, the highest dose tested, the AUC for disease score was reduced 79% (Supplementary Figure 5A). Histological analyses were performed on hind paws. JNJ-54271074 treatment at 60 mg/kg BID significantly reduced the total histopathology score by 80% (Fig. 5B), and significantly reduced inflammation, cartilage damage and bone destruction compared with vehicle (Supplementary Figure 5B). Analysis of inflammatory cells from hind paws by flow cytometry demonstrated that the infiltrated T cells (CD45^+^ CD3^+^) lacked CD4, CD8 and γδ T cell receptor expression (Fig. 5C and Supplementary Figure 5C). These cells produced IL-17A but little to no IFNγ (Fig. 5C). JNJ-54271074 at 60 mg/kg BID decreased this IL-17A-producing cell population by ~70% (Fig. 5D).

### Inhibition of IL-17A production by stimulated PBMC from rheumatoid arthritis patients

**Figure 6:**
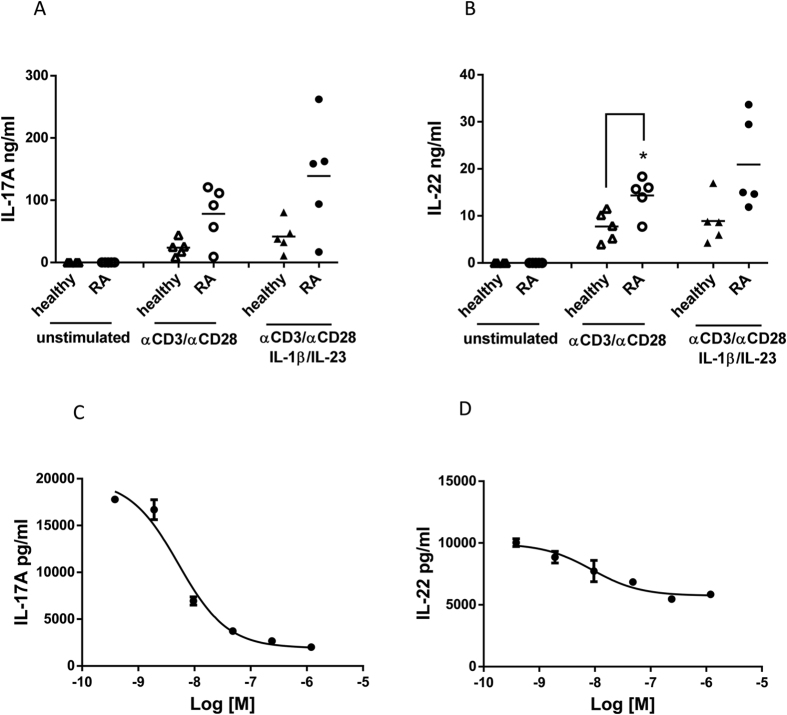
IL-17A production from PBMC of healthy donors and RA patients and effect of JNJ-54271074 on IL-17A and IL-22 production from RA PBMCs. (**A-B**) Comparison of accumulated IL-17A after 3 days of culture. PBMCs were stimulated with anti-CD3/anti-CD28 only or anti-CD3/anti-CD28 plus IL-1β and IL-23 for 3 days, and supernatants were analyzed for IL-17A and IL-22 using MSD or ELISA. Each symbol represents mean value of duplicate assays from individual healthy donors (n = 5) or RA patients (n = 5). (**C-D**) Representative dose response curves of IL-17A and IL-22 for JNJ-54271074 in RA PBMC for one of five patients. PBMCs were stimulated with anti-CD3/anti-CD28 plus IL-1β and IL-23 for 3 days in the presence of titrated JNJ-54271074. Data are presented as mean ± SD of duplicate assays. The statistical analyses were performed using unpaired t test with Welch’s correction, *P < 0.05; **P < 0.01; ***P < 0.005.

As a translational correlate for the mouse CIA data, RORγt modulation was examined in PBMC cultures from five RA patients and five healthy donors. PBMC were cultured with anti-CD3/anti-CD28 in the presence or absence of IL-1β and IL-23, and concentrations of IL-17A were measured in cell supernatants after 3 days of stimulation. PBMCs from RA patients produced more IL-17A than PBMCs from healthy donors with anti-CD3/anti-CD28 stimulation alone, and IL-17A production was further increased with the addition of IL-1β and IL-23 (Fig. 6A). A similar trend was observed for IL-22 production (Fig. 6B); whereas concentrations of IL-13, a cytokine not considered playing a key role in RA pathogenesis, were not significantly different between RA and healthy donors (Supplementary Figure 6). JNJ-54271074 inhibited IL-17A production from PBMC from RA subjects with IC_50_ of 5 ± 1.2 nM (Fig. 6C), which was similar to the IC_50_ typically observed for this compound in PBMC from healthy donors (IC_50_ = 3 ± 0.9 nM). JNJ-54271074 also demonstrated dose-dependent inhibition of IL-22 production, although the efficacy was lower (44% inhibition) than that observed for IL-17A (88% inhibition) (Fig. 6D).

### Inhibition of IL-23-induced dermal psoriatic-like inflammation in mice

**Figure 7:**
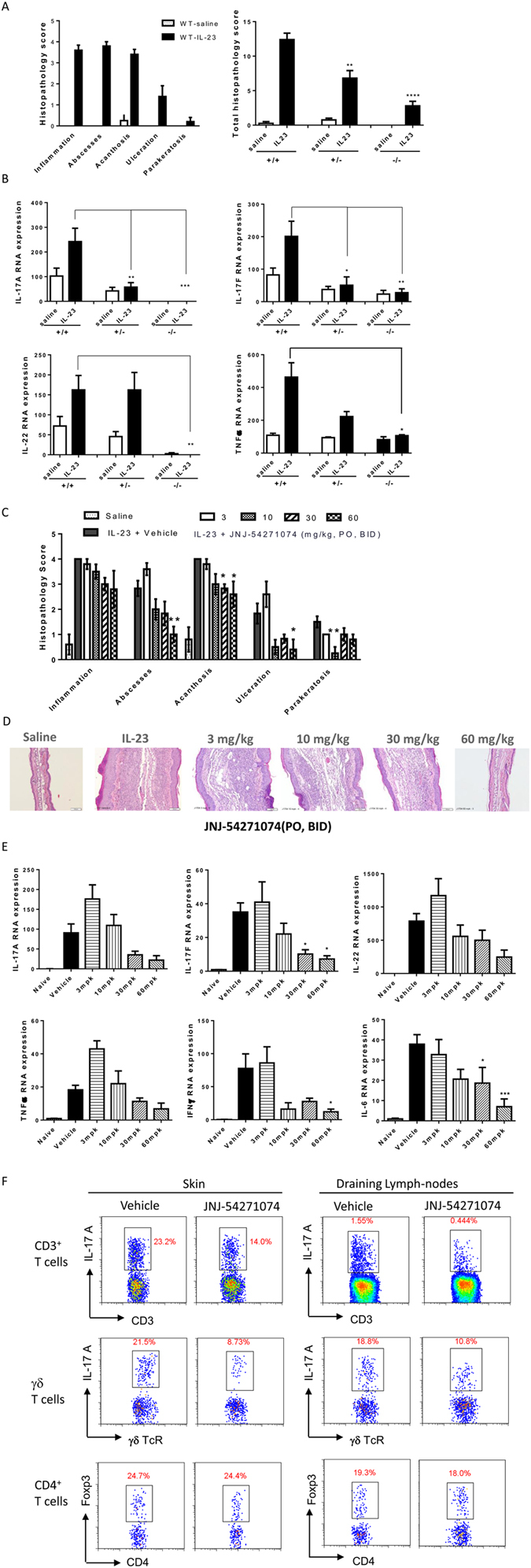
The critical role of RORγt in development of IL-23 induced psoriasis-like mouse model and the inhibitory effect of JNJ-54271074 in this model. (**A**) Individual histopathology scores of skin changes, including inflammation, abscesses, acanthosis, ulceration and parakeratosis in wild-type mice and total score in the ears of wild-type (+/+), RORγt heterozygous (+/−) and RORγt homozygous knockouts (−/−) after saline or IL-23 intradermal injection for 7 days. (**B**) mRNA expression of IL-17A, IL-17F, IL-22 and TNFα in the ears of wild-type, RORγt^+/−^ and RORγt^−/−^. (**C-D**) Individual histopathology score and representative images of skin changes in the ears of IL-23 intradermally injected wild-type mice that were orally dosed with vehicle or JNJ-54271074 at 3, 10, 30 and 60 mg/kg BID for 7 days. (**E**) Dose-dependent inhibition of JNJ-54271074 on mRNA expression of various cytokines in the ear. β2M was used as an endogenous control for RNA quantitation. The relative expression level was calculated based on the formula: 1.8^ (β 2M CT- Target Gene CT) * 10,000^45^. (**F**) Representative flow cytometry analysis of IL-17A-producing cells in the ears and DLNs. Cells extracted from ears or DLNs of 4 to 5 vehicle- or 30 mg/kg JNJ-54271074-treated mice were pooled and stained for IL-17A^+^/CD3^+^ T cells and IL-17A^+^/γδ^+^ T cells. Values are representative of two independent experiments and are presented as the mean ± SEM, n = 4-5 mice per group. Statistical analyses were performed with one-way ANOVA (Dunnett multiple comparisons), *P < 0.05; **P < 0.01; ***P < 0.005; ****P < 0.0001.

Intradermal injection of IL-23 produces skin inflammation in mice that shares histological and transcriptional features of human psoriasis^45^,^46^. We first examined the skin inflammation induced by injecting IL-23 into wild-type mice, and then investigated the role of RORγt in this model by using RORγt deficient, homozygous (RORγt^−/−^) and heterozygous (RORγt^+/−^) mice. IL-23 injection induced skin inflammation in the ears, as demonstrated by increased histology scores of multiple parameters (inflammation, abscesses, acanthosis, ulceration, parakeratosis), as well as mRNA expression levels of IL-17A, IL-17F, IL-22 and TNFα, compared with saline treated mice (Fig. 7A and B). In contrast, RORγt^−/−^ mice were almost completely protected from IL-23-induced effects with minimal histological or RNA changes compared with saline treated mice. RORγt^+/−^ mice showed attenuated inflammation in response to IL-23 injection compared with wild type mice. T cell infiltration in the ear injected with IL-23 was also investigated by flow cytometry. CD3^+^ T cells were undetectable after saline injection (data not shown). WT mouse ears injected with IL-23 contained 22% IL-17A^+^ γδ^+^ T cells and 4.6% IL-17A^+^ CD4^+^ T cells when gated on CD3^+^ cells. In IL-23 injected ears of RORγt^+/−^ mice, IL-17A^+^/γδ^+^ T cells were undetectable and IL-17A^+^/CD4^+^ T cells were reduced by 50% (Supplementary Figure 7A). In RORγt^−/−^ mice, IL-17A-producing CD4^+^ and γδ^+^ T cells were not detectable after IL-23 injection. Next we tested the activity of JNJ-54271074 in this model. JNJ-54271074 was administered orally at several doses to mice, and the inflammatory response to the IL-23 challenge was examined. JNJ-54271074 decreased skin inflammation indicated by reduction of total skin histology scores and significant decrease of skin abscesses and acanthosis, in a dose-dependent manner (Fig. 7C and Supplementary Figure 7B). The dose-dependent effect of JNJ-54271074 was clearly shown by the decreased skin thickness in histology images (Fig. 7D). At the RNA level, JNJ-54271074 significantly inhibited expression of IL-17F, IFNγ and IL-6, and at 30 and 60 mg/kg suppressed IL-17A, IL-22 and TNFα although these effects did not reach statistical significance (Fig. 7E). We observed an increase in IL-17A and TNFα mRNA expression at the lowest dose (3 mg/kg) of JNJ-54271074, which may be due to inter-animal biological variation, and was not observed for other cytokines. Infiltrated cells in the ear skin and draining lymph nodes were analyzed by flow cytometry, showing that both IL-17A-producing total CD3^+^ T cells and IL-17A-producing γδ^+^ T cells were reduced by 40% and 60% respectively in ear skin, and 71% and 43% respectively in DLNs, after dosing 30 mg/kg BID. The FOXP3^+^ CD4^+^ T cells were also analyzed in these tissues and the populations are very similar between vehicle and compound treated groups (Fig. 7F).

### Effect on cytokine production induced by streptococcal extract in the co-culture of memory (CLA^+^/CLA^−^) T cells and autologous epidermal cells from guttate psoriasis patients

**Figure 8:**
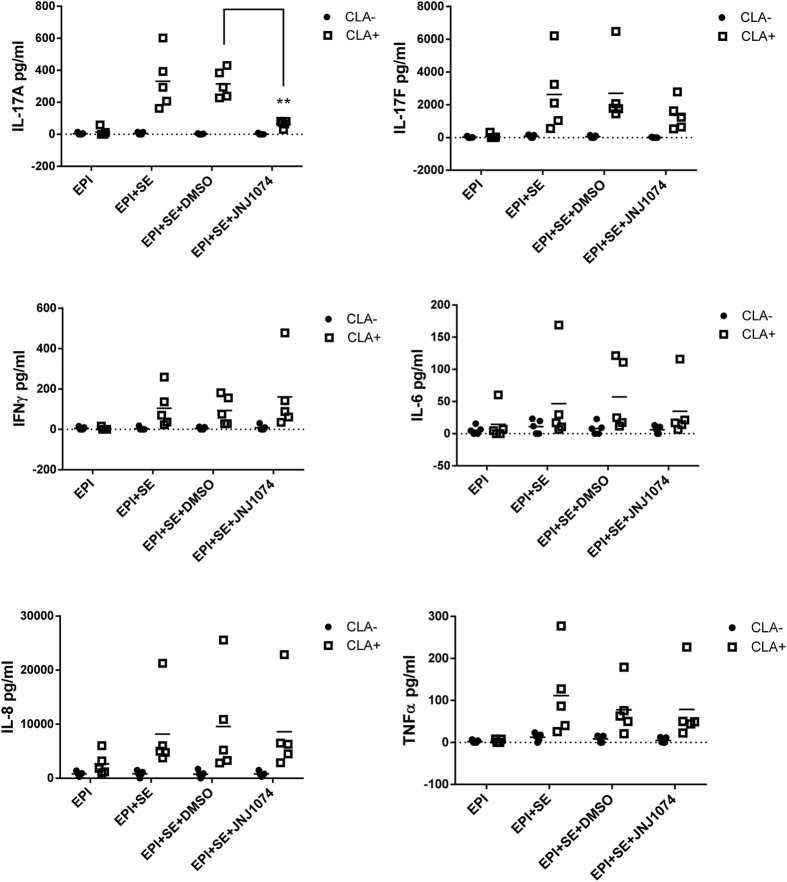
Streptococcal extract (SE)-induced cytokine production in the co-culture of CLA^+^ or CLA^−^ T cells with autologous epidermal cells from guttate psoriasis patients. The cells were cultured for 5 days in the absence or presence of 1 μM JNJ-54271074. Accumulated cytokines (IL-17A, IL-17F, IFNγ, IL-6, IL-8 and TNFα) in supernatants were quantified by multiplex fluorescent bead-based immunoassay. Data are pooled and presented as mean values ± SD of five independent experiments, one patient per experiment. Statistical analyses were performed using unpaired t test with Welch’s correction, *P < 0.05; **P < 0.01; ***P < 0.005.

JNJ-54271074 was next investigated in a human *ex vivo* translational model in which streptococcal extract (SE) triggers psoriatic responses in a co-culture of epidermal cells and skin homing CLA^+^ T cells obtained from guttate psoriasis patients^35^. SE was found to preferentially induce activation of CLA^+^ skin homing T cells, but not CLA^−^ T cells, in co-culture with autologous epidermal cells, as indicated by much higher level of IL-17A, IL-17F, IFNγ, IL-6, TNFα and IL-8 in CLA^+^ versus CLA^−^ T cells (Fig. 8). In SE-stimulated CLA^+^/epidermal cell co-culture, JNJ-54271074 at 1 μM decreased IL-17A production significantly by 78%, in comparison to DMSO control. IL-17F production was reduced by 40%, and IL-6 was reduced by 32%, but these difference were not statistically significant. Conversely, JNJ-54271074 increased the amount of IFNγ production by 64%, although this did not reach statistical significance. JNJ-54271074 had no effect on TNFα and IL-8 production, indicating the specificity of the effects on IL-17A and IL-17F.

## Discussion

RORγt plays a critical role in driving Th17 cell differentiation and expansion, as well as IL-17 production in innate and adaptive immune cells, making it an attractive therapeutic target for modulating diseases associated with the IL-23/IL-17 pathway. In this paper, we report the activity of a novel, highly-selective, RORγt inverse agonist, JNJ-54271074, that inhibits RORγt function through blocking the binding with co-activator while promoting the recruitment of co-repressor, translating to potent and efficacious inhibition of RORγt-driven transcription in primary immune cells. Several other RORγt small-molecule modulators have been shown to block Th17 cell differentiation and IL-17A production, as well as demonstrate efficacy in EAE, CIA or imiquimod-induced psoriasis models^47^,^48^,^49^,^50^,^51^,^52^,^53^. We show in this paper for the first time, that deficiency or pharmacological modulation of RORγt significantly inhibited skin inflammation and expression of cytokine genes induced by IL-23, in a mouse psoriasis-like model. Additionally, oral dosing of JNJ-54271074 during the effector phase of mouse CIA dose-dependently decreased arthritic score, and histological inflammation, cartilage and bone damage. We furthermore made the translational links to human RORγt modulation by demonstrating that JNJ-54271074 had a significant impact on circulating and skin-homing human memory Th17 cells from RA and psoriasis patients, respectively.

JNJ-54271074 blocked differentiation of naive CD4^+^ T cells to Th17 cells and suppressed IL-17A production by *in vivo* differentiated effector/memory Th17 cells, the major pathogenic cell population in several inflammatory diseases. Among the cytokines produced by Th17 cells, IL-17A is the most significantly inhibited by JNJ-54270174, indicating that RORγt has a highly specific regulatory footprint in human Th17 cells, similar to what has been reported for mouse Th17 cells by Ciofani *et al*.^54^.

In contrast to the effects on IL-17 producing cells, modulation of RORγt by JNJ-54271074 had minimal impact on Th1 or Treg cells. No inhibition or slight increase of Th1 cell differentiation was observed, based on IFNγ production or percentage of IFNγ^+^ cells. Importantly, JNJ-54271074 did not suppress Treg differentiation as measured by percentage of FOXP3^+^ cells, slightly increased RNA expression level of FOXP3, and did not affect Treg function measured as suppressive activity on effector T cell proliferation. Together, our *in vitro* data suggest that modulation of RORγt does not compromise the functions of Th1 and Treg cells, but specifically decreases Th17 cell activities. In addition, in human CCR6^+^ T cell activation studies, JNJ-54271074 slightly increased IL-10 production at concentrations where IL-17A production was 80–90% inhibited, consistent with a mild increase in FOXP3 expression in compound-treated CD4^+^ T cells under Treg polarization conditions. Our demonstration of increased IL-10 protein production through inhibition of RORγt in human T cells is consistent with results of previous mouse studies, where IL-10 RNA expression was significantly up-regulated in RORγt-deficient or inhibitor-treated Th17 cells^51^,^52^.

In addition to Th17 cells, IL-17A is produced by innate lymphoid cells such as γδ T cells and NKT cells^43^,^44^,^55^. In psoriasis patients, γδ T cells were reported to be increased in lesional skin and to produce large amounts of IL-17A^55^. Increased subsets of NKT cells in lesional skin, and correlation of changes in their presence with efficacy of psoriasis treatments, have also been reported^56^. Our *in vitro* studies demonstrated that, in addition to its inhibitory effects on Th17 cells, JNJ-54271074 blocked IL-23-induced IL-17A production from both mouse and human γδ T cells and NKT cells. We also found that IL-23 alone was able to induce cytokine production from mouse splenocytes, and that JNJ-54271074 showed dose-dependent inhibition of IL-23-induced production of IL-17A, IL-17F and IL-22. Together these data show that JNJ-54271074 blocks IL-23-induced cytokine production from multiple immune cell types.

It has been reported that IL-17A is highly expressed in RA synovium^57^,^58^ and in the synovial fluid of patients with early RA^59^. An increased population of CCR6^+^ memory T cells, expressing high levels of IL-17A and TNFα, was identified in the peripheral blood of patients with RA^60^. We tested the effects of JNJ-54271074 on joint pathology in the mouse CIA model and in a human translational correlate, using PBMCs from RA subjects. In our CIA studies, oral dosing of JNJ-54271074 significantly attenuated inflammation, and achieved maximum inhibition of 78% of final arthritic score, and 80% of total histopathology score, at a dose of 60 mg/kg, BID. Another RORγt inverse agonist, SR2211, was reported to reduce joint inflammation, along with improvement in clinical scores, in a mouse CIA model^49^. Analysis of cells in the inflamed paws in our study revealed that ~50% CD3^+^ T cells produced IL-17A, and that this population was significantly inhibited by JNJ-54271074. In addition, we found these cells did not express CD4, CD8, nor γδ T cell receptors, thus resembling RORγt^+^ CD3^+^ CD4^−^CD8^−^ entheseal resident T cells, reported to be induced by IL-23 in a mouse spondyloarthropathy model^61^. Beyond the mouse arthritis model, we also investigated the effects of JNJ-54271074 in human PBMC from RA patients. Under neutral activation conditions, CD4^+^ T cells from these patients produced more IL-17A than PBMCs from healthy subjects, and this difference was more pronounced in the presence of IL-1β and IL-23. JNJ-54271074 dose-dependently inhibited IL-17A production in PBMCs from RA patients. Taken together, our data from both rodent and human studies support the modulation of RORγt as a potential therapeutic in RA, psoriatic arthritis or ankylosing spondylitis. Interestingly, biologics targeting IL-17A or IL-17RA have had varied success in treating RA^62^,^63^,^64^,^65^, but are showing promise in psoriatic arthritis^32^,^66^. Targeting the IL-12/23 pathway is also beneficial in psoriatic arthritis^67^,^68^, and it is possible that RORγt modulation will have effects beyond blockade of IL-17A or IL-17RA in inflammatory arthritis indications.

While the role of the IL-23 and IL-17 pathways is evolving in inflammatory arthritis indications, their role in the pathogenesis of plaque psoriasis is clearly validated. We used an IL-23-induced mouse skin inflammation model to investigate for the first time the role of RORγt modulation. This model produces skin inflammation in mice that shares many histological features of human psoriasis^45^ and transcriptome analysis has revealed that, among several mouse psoriasis models, the IL-23 model best matches expression patterns in human psoriatic lesions^46^. Intradermal injection of IL-23 into mouse skin induced mRNA expression of inflammatory cytokines including IL-17A, IL-17F, IL-22, IFNγ, TNFα and IL-6, and was accompanied by infiltration of immune cells into the skin, culminating in histopathological changes^69^. Compared to wild type mice, RORγt-deficient homozygous (−/−) and heterozygous (+/−) mice showed abolished or attenuated changes in cytokine mRNA and skin histopathology in response to intradermal IL-23. JNJ-54271074 treatment also decreased cytokine mRNA expression, histopathology and dermal T cell populations in this IL-23-dependent, psoriasis-like skin inflammation model. Our data with JNJ-54271074 were similar to data published with another RORγt modulator in a different psoriasis-like mouse skin inflammation model, induced by the TLR7 agonist, imiquimod^50^. In addition, in our IL-23 model, Treg population in skin and draining lymph nodes was not affected after 7-day compound treatment, consistent with our early discussion that JNJ-54271074 does not affect Treg. We also investigated the effects of JNJ-54271074 in an *ex vivo* translational model utilizing circulating memory CLA^+^ (skin-homing) T cells and autologous epidermal cells from psoriasis patients, upon activation with an extract of *Streptococcus pyogenes*, a clinically relevant trigger of psoriasis. RORγt modulation by JNJ-54291074 caused significant inhibition of IL-17A and decreased IL-17F production in this human cell co-culture model. This effect of JNJ-54271074 on IL-17A and IL-17F production is consistent with the gene expression data from the IL-23-induced psoriasis-like mouse model. In addition, it is interesting to note that gene expression analyses of psoriatic skin after 12 weeks of anti-IL-23 mAb guselkumab treatment also showed significant inhibition of both IL-17A and IL-17F gene expression^70^. Together the data from the IL-23-induced skin inflammation mouse model and this human primary cell co-culture model provide support for a beneficial role for RORγt modulation in treating human psoriasis.

In summary, JNJ-54271074 has demonstrated robust pharmacological inhibition of the IL-23 and IL-17 pathways, both *in vitro* and *in vivo*. Biologics blocking IL-23 and IL-17 pathways have resulted in significant efficacy in treating psoriasis^47^. IL-12/23 inhibition is also efficacious in the treatment of psoriatic arthritis, and emerging clinical data are promising for IL-17 pathway inhibitors in this indication as well^71^. Our *in vivo* mouse model data and human translational data demonstrate that modulation of RORγt function, using molecules such as JNJ-54271074, holds promise as a novel oral therapeutic approach for the treatment of psoriasis and psoriatic arthritis.

## Additional Information

**How to cite this article**: Xue, X. *et al*. Pharmacologic modulation of RORγt translates to efficacy in preclinical and translational models of psoriasis and inflammatory arthritis. *Sci. Rep.* **6**, 37977; doi: 10.1038/srep37977 (2016).

**Publisher's note:** Springer Nature remains neutral with regard to jurisdictional claims in published maps and institutional affiliations.

## Supplementary Material

Supplementary Information
