# Supplementary material for: Pharmacologic modulation of RORγt translates to efficacy in preclinical and translational models of psoriasis and inflammatory arthritis
Source: Sci Rep. 2016 Dec 1;6:37977. doi: 10.1038/srep37977 (PMC5131364; doi:10.1038/srep37977)

Pharmacologic modulation of RORγt translates to efficacy in preclinical and translational models of psoriasis and inflammatory arthritis

Xiaohua Xue[1], Pejman Soroosh[1], Aimee De Leon-Tabaldo[1], Rosa Luna-Roman[1], Marciano Sablad[1], Natasha Rozenkrants[1], Jingxue Yu[1], Glenda Castro[1], Homayon Banie[1], Wai-Ping Fung-Leung[1], Luis Santamaria-Babi[3], Thomas Schlueter[4], 'Michael Albers[4], Kristi Leonard[2], Alison L Budelsky[1], Anne M Fourie[1§]

[§]Correspondence to A.M.F. (email: Afourie@its.jnj.com)

Supplementary Methods

Mouse $CD4^+$ T cell *in vitro* Th1 differentiation

Total $CD4^+$ T cells were isolated from splenocytes of C57/BL6 mice (Chareles River Labrotory) using a $CD4^+$ T cell isolation kit (Miltenyl Biotec, Auburn, CA), following the manufacturer's instructions. Cells were resuspended in culture medium (described in Method section of Th17 differentiation) and were added to 96-well plates at $2x10^5$ per well. Titrated JNJ-54271074 was added to each well at final DMSO concentration of 0.2%. Cells were incubated for 1 hour, after which Th1 cell differentiation medium was added with final concentrations of 1 μg/ml anti-CD3, 5 μg/mL anti-CD28 (BD Pharmingen, San Diego, CA), 10 μg/ml anti-IL4, 10 ng/mL IL-12, (R&D Systems, Minneapolis, MN). Cells were cultured at 37 ^oC and 5% CO_2 for 3 days. Supernatants were collected and the accumulated cytokines in culture were measured by ELISA kits (R&D Systems, Minneapolis, MN) following manufacturers' instructions.

IL-23 induced IL-17A and IL-22 production in mouse splenocytes

splenocytes were isolated from C57/BL6 mice and resuspended in medium and cultured in 96-well plates at $2.5x10^5$ per 100 μL per well. Titrated JNJ-54271074 in DMSO were added into each well and cells were incubated for 1 hour, then medium containing IL-23 and IL-2 (R&D systems) was added to each well at final concentration of 1 ng/mL and 10 U/ml, respectively. Cells were cultured at 37 ^oC and 5% CO_2 for 4 days. Supernatants were collected and the accumulated cytokines in culture were measured by ELISA according to the manufacturer's instructions (IL-17A kit from R&D systems, and IL-22 from Biolegend San Diego).

Human $CCR6^+$ cells *in vitro* culture without activation or under Th1 differentiation condition

Human $CCR6^+$ cells were isolated as described in Method. For experiment without activation, cells were cultured with recombinant IL-7 (R&D) at final concentration of 10 ng/ml in the presence of 1 μM JNJ-54271074 for 4 days before intracellular staining. For Th1 differentiation, cells were cultured in a 96-well plate pre-coated with 1 μg/ml anti-CD3 (BD Bioscience) at 1×10^5 cells per well. Titrated JNJ-54271074 was added to each well at final DMSO concentration of 0.2%, followed by addition of anti-CD28 (eBioscience, San Diego CA) at final concentration of 5 μg/ml, anti-IL-4 at final concentration of 10 μg/ml, and IL-12 at final concentration of 10 ng/ml. Cells were cultured at 37 ^{o}C and 5% CO2 for 6 days. Supernatants were assayed for accumulated cytokines using MSD and cells were used for intracellular staining.

Human Treg *in vitro* differentiation

Total $CD4^+$ T cells were isolated from blood of healthy donor using $CD4^+$ T cell isolation kit II (Miltenyl Biotec, Auburn, CA), and cultured in medium (IMDM supplemented with 10% SRF (knockout serum replacement, Life technologies), 1 mM sodium pyruvate, 1X Non-essential amino acids , 100 IU Penicillin and 100 ug/mL streptomycin) in 96-well plate at 4 $x10^5$ cells per well. DMSO or 1 μM JNJ-54271074 was added to the culture and incubated for one hour, then cells were cultured either under Th0 condition (anti-CD3/CD28 beads only, $2x10^6$/ml prepared using human T cell activation/expansion kit, Miltenyl Biotec) or Treg differentiation condition with the final concentration of anti-CD3/CD28 beads, 200U/ml IL-2, 10 ng/ml TGFβ1, 10 μg/ml anti-IL-4, 10 μg/ml anti-IFNγ, 10 μg/ml anti-IL-12. Cells were cultured at

37 ^{o}C and 5% CO_2 for 6 days. Cells were used for flow cytometry analysis of $FOXP3^{+}CD4^{+}$ T cells and RNA isolation to determine FOXP3 expression. The probe sets were purchased form Life technologies, FOXP3 HS01085834_m1, GAPDH HS02758991_g1.

Human inducible regulatory T cells generation and suppressive function

To generate inducible regulatory T cells, naïve CD4 T cells was purified from PBMCs using naive $CD4^{+}$ T cell isolation kit II, human (Miltenyi) and stimulated with anti-CD3/anti-CD28 antibodies plus IL-2 and TGF-β (10 ng/ml each, R&D system) in the presence or absence of 1μM JNJ-54271074 . After 6-7 days T cells were washed and Foxp3 expression was evaluated by FACS. To evaluate the suppressive function, iTreg were co-cultured with Carboxyfluorescein succinimidyl ester (CFSE, eBioscince) labeled total $CD4^{+}$ T cells (responder T cells from same donor) in 1:2 ratio and anti-CD3/CD28 microbeads (Miltenyi) added to activate responder T cells. After 4 days CFSE dilution was evaluated by FACS as an indication of responder T cell's proliferation.

Supplementary Legend

Supplementary Figure 1. Dose-dependent inhibition on mouse IL-17A production and minimum effect on mouse IFNγ production. $CD4^{+}$ T cells were isolated from mouse spleen and cultured under Th17 (A) or Th1 (B) polarization condition for 3 days in the absence or presence of titrated JNJ-54271074. Accumulated IL-17A or IFNγ was measured by ELISA.

Supplementary Figure 2. Dose-dependent inhibition of JNJ-54271074 on IL-23-induced production of IL-17A and IL-22 in mouse splenocytes. Total splenocytes were cultured with IL-23 and IL-2 in the presence of titrated JNJ-54271074 for 4 days, and supernatants were measured for IL-17A and IL-22 by ELISA. IC_{50} was determined using Prism Graphpad.

Supplementary Figure 3. Effect of JNJ-54271074 on $IFN\gamma^{+}/CD4^{+}$ population and IFNγ production in human $CCR6^{+}$ T cells. $CCR6^{+}$ T cells were purified from blood of healthy donor, and cultured with IL-7 in the presence or absence of JNJ-54271074 for 4 days. Cells were analyzed by flow cytometry for $17A^{+}$ and IL-$17A^{+}/22^{+}$ population (A). $CCR6^{+}$ T cells were cultured under Th1 polarization condition in the absence or presence of titrated JNJ-54271074 for 6 days. Cells from DMSO or 1 μM JNJ-54271074 treated wells were analyzed by flow cytometry for $IFN\gamma^{+}$ and IL-$17A^{+}$ population (B) and IFNγ level in supernatants was measured by MSD (C).

Supplementary Figure 4. *In vitro* Treg differentiation and effect of JNJ-54271074 on Treg differentiation and FOXP3 expression, and suppressive activity of iTreg. (A) Flow cytometry analysis of $FOXP3^{+}/CD4^{+}$. $CD4^{+}$ T cells were isolated from blood of healthy donor and cultured in IMDM/10% SRF (Knockout Serum replacement, Life technologies) under Th0 or Treg differentiation condition for 6 days. DMSO or 1 μM JNJ-54271074 was added to the culture. (B) RNA expression of FOXP3. On day 6, RNA was extracted from the cells and expression level of FOXP3 was measured by RT-PCR. GAPDH was used as endogenous control for RNA quantitation and calculated based on the formula: 1.8^(GAPDH CT- Target Gene CT) * 10,000. (C) Inducible Tregs were generated from naïve CD4 T cells in the presence of compounds (1 μM)

or vehicle. After 7 days Tregs were collected, washed and co-cultured with CFSE labeled responder CD4 T cells in 1:2 ratio. Anti-CD3 and anti-CD28 were added to stimulate $CD4^+$ T cells. Four days later CFSE dilution was analyzed by FACS.

Supplementary Figure 5. (A) AUC of clinical score of CIA following treatment with vehicle or JNJ-54271074 (0.3, 3, 10, 30 and 60 mg/kg BID). Values are the mean ± SEM. **= p<0.01, ***= p<0.005 versus vehicle by one way ANOVA for time course and two way ANOVA for AUC (Dunnett's multiple comparisons test). (B) Histopathology score of inflammation, cartilage damage, bone destruction and remodeling in hind paws, harvested on day 35 (n= 14-18 paws per group). Values are the mean ± SEM. *= p<0.05 versus vehicle by one way ANOVA (Bonferroni's Multiple Comparison Test). (C) Flow cytometry analysis on $CD3^+/\gamma\delta^+$ T cell population in front paws on day 35.

Supplementary Figure 6. (A) Comparable level of IL-13 production in PBMCs of healthy donor and RA patient. PBMCs were stimulated with anti-CD3/anti-CD28 only or anti-CD3/anti-CD28 plus IL-1β and IL-23 for 3 days, and supernatants were analyzed for IL-13 using MSD.

Supplementary Figure 7. (A) Flow cytometry analysis of infiltrated T cells in the ear. Cells extracted from ears of wild-type, ROR$\gamma t^{+/-}$ mice or ROR$\gamma t^{-/-}$ were pooled and stained for CD45, CD3, CD4, $\gamma\delta$ T and IL-17A. Total $CD45^+CD3^+$ T cells were gated then IL-17$A^+CD4^+$and IL-17$A^+\gamma\delta^+$ T cells were quantitated. (B) Total histopathology score in the ears of IL-23 intradermally injected wild-type mice that were orally dosed with vehicle or JNJ-54271074 at 3, 10, 30 and 60 mg/kg BID for 7 days.

Supplementary Figure 1

A B

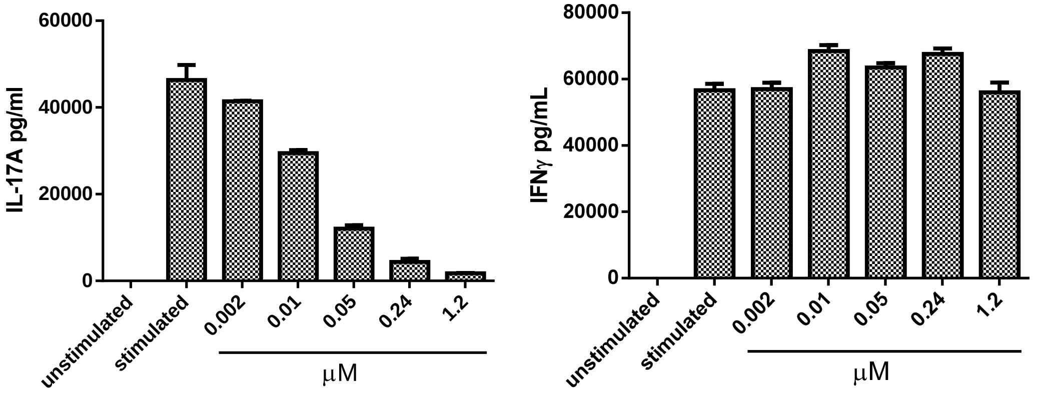

Supplementary Figure 2

A **B**

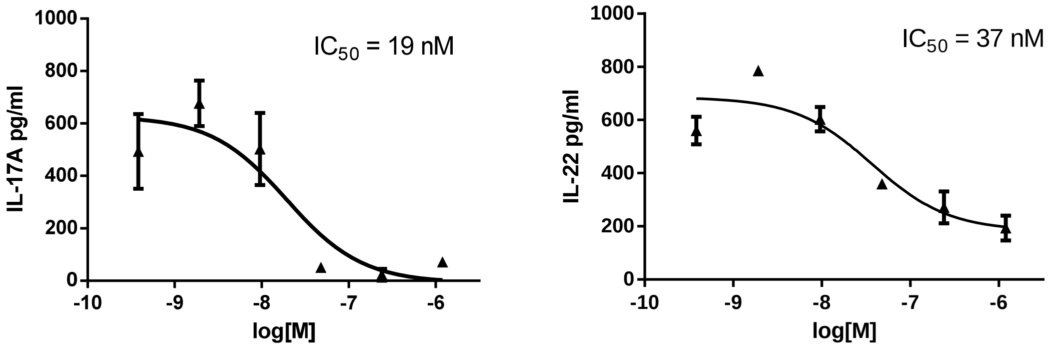

Supplementary Figure 3

A

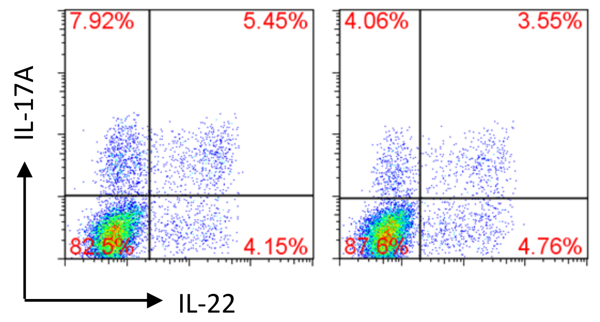

B

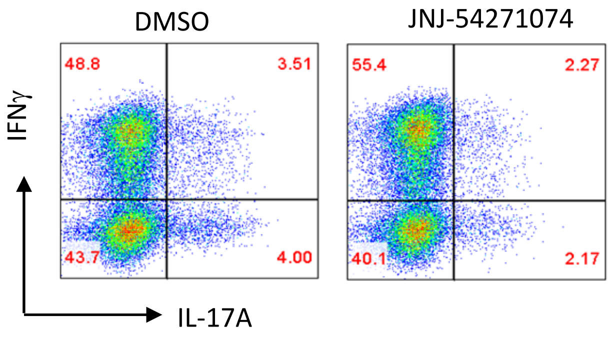

C

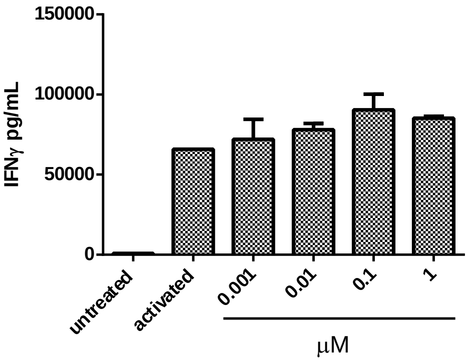

Supplementary Figure 4

A

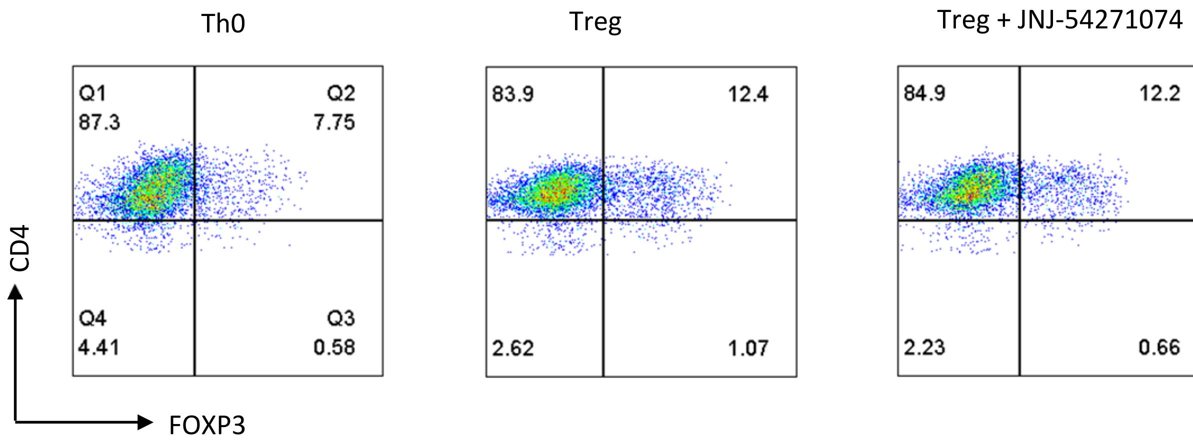

B

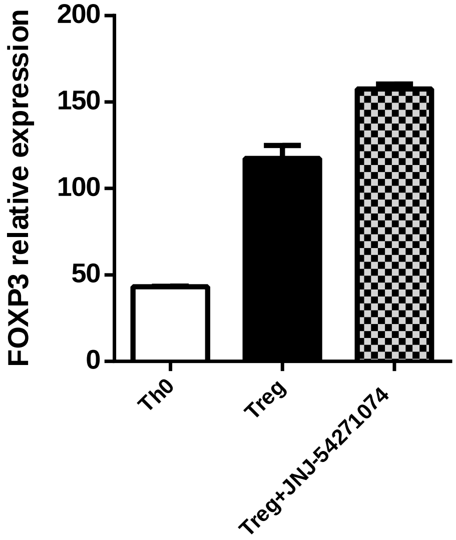

C

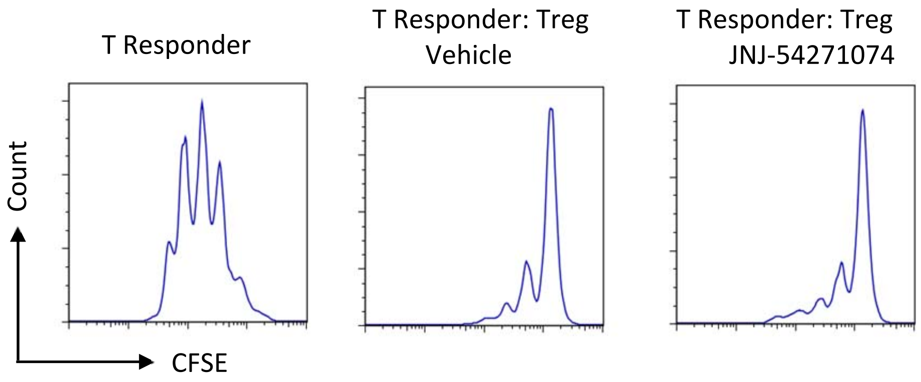

Supplementary Figure 5

A

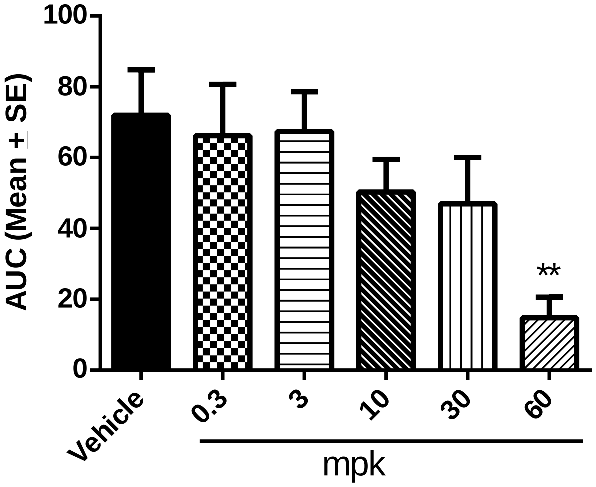

B

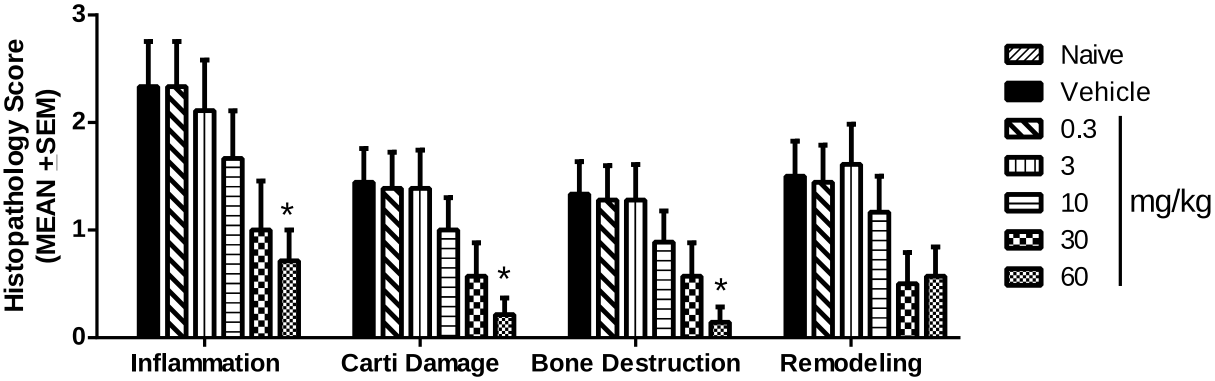

C

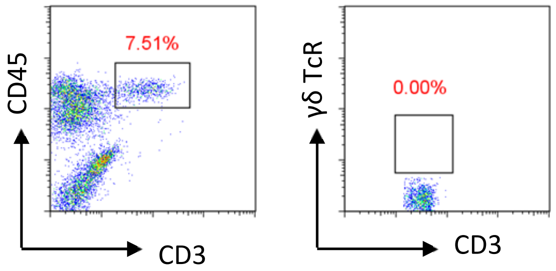

Supplementary Figure 6

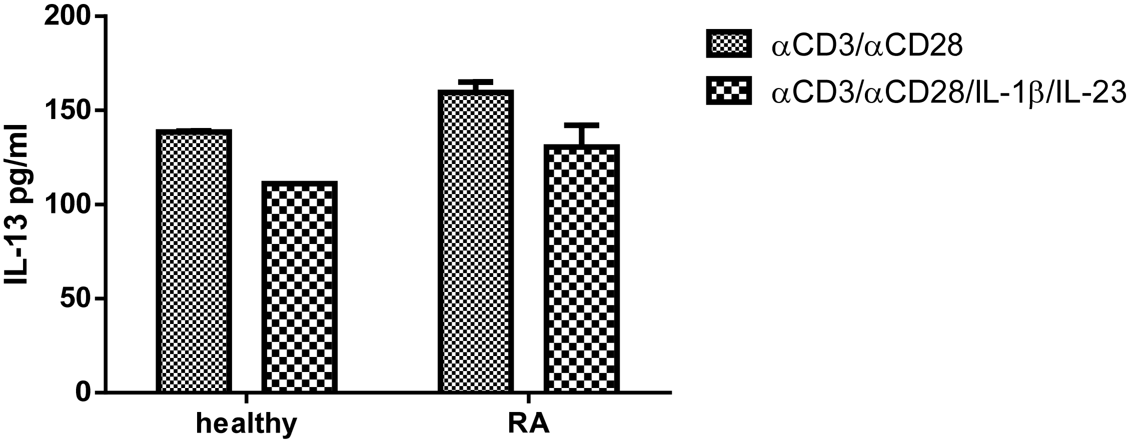

Supplementary Figure 7

A

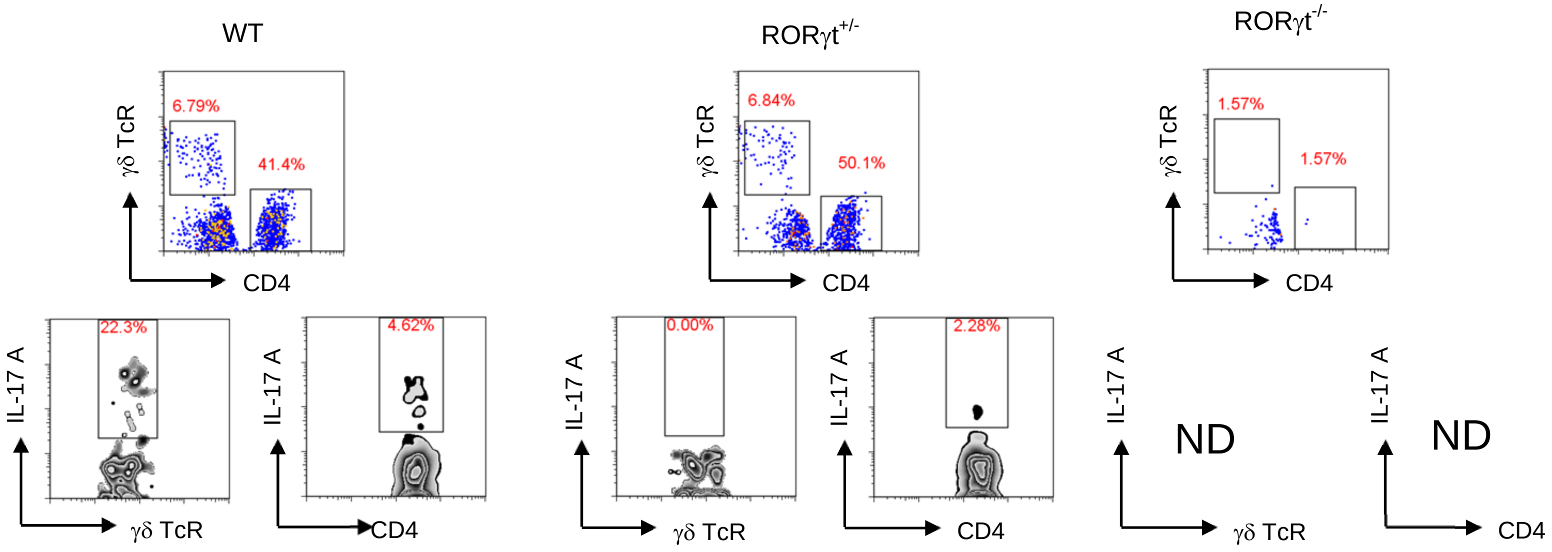

B

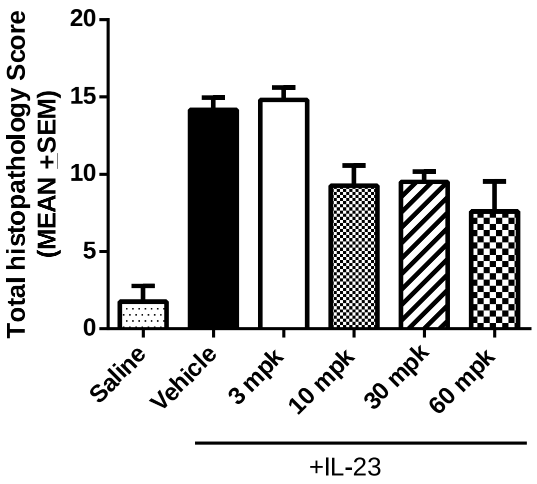

Supplement: Supplementary Information [file srep37977-s1.pdf]
